# Mechanisms of NMDA Receptor Inhibition by Biguanide Compounds

**DOI:** 10.3390/ph17091234

**Published:** 2024-09-19

**Authors:** Arseniy S. Zhigulin, Anastasiya O. Novikova, Oleg I. Barygin

**Affiliations:** Sechenov Institute of Evolutionary Physiology and Biochemistry of RAS, 44, Toreza Prospekt, Saint Petersburg 194223, Russia; arseniy.zhigulin@yandex.ru (A.S.Z.); novikovaanastasianao@gmail.com (A.O.N.)

**Keywords:** NMDA receptors, pharmacological modulation, patch clamp, biguanides, proguanil, cycloguanil, phenformin, antagonsits

## Abstract

N-methyl-D-aspartate (NMDA) receptors are inhibited by many medicinal drugs. The recent successful repurposing of NMDA receptor antagonists ketamine and dextromethorphan for the treatment of major depressive disorder further enhanced the interest in this field. In this work, we performed a screening for the activity against native NMDA receptors of rat CA1 hippocampal pyramidal neurons among biguanide compounds using the whole-cell patch-clamp method. Antimalarial biguanides proguanil and cycloguanil, as well as hypoglycemic biguanide phenformin, inhibited them in micromolar concentrations, while another hypoglycemic biguanide metformin and antiviral biguanide moroxydine were practically ineffective. IC_50_ values at −80 mV holding voltage were 3.4 ± 0.6 µM for cycloguanil, 9.0 ± 2.2 µM for proguanil and 13 ± 1 µM for phenformin. The inhibition by all three compounds was not competitive. Cycloguanil acted as an NMDA receptor voltage-dependent trapping channel blocker, while proguanil and phenformin acted as allosteric inhibitors. Our results support the potential clinical repurposing of biguanide compounds for the treatment of neurodegenerative disorders linked to glutamatergic excitotoxicity while also providing a better understanding of structural determinants of NMDA receptor antagonism by biguanides.

## 1. Introduction

N-methyl-D-aspartate (NMDA) receptors are a type of ionotropic glutamate receptors that are widely found throughout the nervous system, where they play essential roles in synaptic transmission, synaptic plasticity, learning, memory and numerous other processes [1]. Their overactivation, leading to excessive calcium entry and excitotoxicity, is involved in many pathologies, including Alzheimer’s and Parkinson’s diseases and glaucoma [2]. Thus, it is not surprising that several clinically used drugs inhibit NMDA receptors as a primary target. Adamantane derivative memantine has been used for the treatment of Alzheimer’s disease for about 20 years [3]. Recently, ketamine and dextromethorphan (in combination with bupropion) were approved for the treatment of major depressive disorder [4,5]. These are two examples of successfully repurposed compounds. Indeed, ketamine was initially used as a dissociative anesthetic [6] and dextromethorphan as an antitussive agent [7]. The strategy of repurposing already used medicinal drugs for new diseases has gained much attention over the last decades [8], including the fields of neurodegenerative diseases [9,10] and depression [11,12]. This strategy has significant advances compared to the traditional development of new substances. The first and probably the most important one is the knowledge that repositioned drugs are relatively safe in preclinical and clinical settings. This fact allows for reducing the time and investments needed for drug development because expensive preclinical testing and safety assessments has already been performed. Thus, the repositioning strategy has a lower risk of development failure [8].

There are three major classes of NMDA receptor antagonists: competitive antagonists, allosteric antagonists and channel blockers. Competitive antagonists bind to the same sites on NMDA receptor agonist-binding domains as agonists—glutamate on GluN2 subunits and glycine or D-serine on GluN1 subunits [1]. A classic example of a competitive antagonist preventing glutamate binding to GluN2 subunits is D-(−)-2-Amino-5-phosphonopentanoic acid (D-AP5). The inhibition by competitive antagonists can be overcome by the increase in agonist concentration, and none of them are thus used in clinical practice. The second class is allosteric antagonists, which bind to sites different from those abovementioned orthosteric ligands. The majority of the NMDA receptor allosteric antagonists bind to the amino-terminal domains of the receptor. Probably the most well-studied representatives of this class are GluN2B-selective negative allosteric modulators, such as ifenprodil, Ro 25-6981 and EVT-101 [13,14]. They demonstrated neuroprotective properties but did not successfully get through clinical trials because of the rather high risk of side effects [14]. Finally, the third class is channel blockers that bind in the channel pore, physically occluding the ion flux. The action of charged channel blockers is characterized by voltage dependence because their binding sites are situated within the membrane electric field. Channel blockers, in turn, are divided into two subclasses by their interaction with the channel gate: “foot-in-the-door” and trapping blockers [15]. The latter can remain trapped in the channel pore after channel closure. As a result, trapping blockers accumulate in the closed channels and can more effectively inhibit the peak of NMDA receptor-mediated currents. “Foot-in-the-door” blockers, in contrast, prevent channel closure.

Clinically used NMDA receptor channel blockers—memantine, ketamine and dextromethorphan—all contain positively charged nitrogen atoms, and all of them act as moderate affinity trapping channel blockers of NMDA receptors with relatively fast kinetics [16,17]. These properties, to a large extent, determine their clinical tolerability [18], making the studies of molecular mechanisms of inhibition extremely important. Indeed, many other NMDA receptor antagonists are not used in clinical practice because of serious side effects [19,20].

Biguanides are compounds in which two guanidine moieties are fused to form a p-conjugated system [21]. They are used as hypoglycemic, antimalarial and antiviral agents. The simplest biguanide metformin is a first-line medication to treat type 2 diabetes [22]. Phenformin is another hypoglycemic biguanide that was used for diabetes treatment but was withdrawn from the market because of the high risk of fatal lactic acidosis [22]. Despite these risks, the research on phenformin is still ongoing, and it is now considered a drug candidate for several types of cancer because of its antiproliferative effects [23]. Proguanil, yet another biguanide compound, is used to treat and prevent malaria [21]. Its active metabolite, cycloguanil, also belongs to biguanides. Finally, adamantane biguanide moroxydine has antiviral properties against influenza and different other viruses [21]. Nowadays, it is mostly used in veterinary medicine.

Many amidine- and guanidine-containing compounds, including pentamidine, diminazene, furamidine, nafamostat and sepimostat, inhibit NMDA receptors by different molecular mechanisms [24,25,26,27,28]. Taking into account the structural similarity between amidines, guanidines and biguanides, we decided to perform a screening test for activity against NMDA receptors among the five abovementioned representatives of the latter group. To the best of our knowledge, among biguanide compounds, only phenformin was shown to inhibit NMDA receptors, and molecular mechanisms of its action were not investigated [29]. We confirmed the inhibitory action of hypoglycemic biguanide compound phenformin in our experiments and showed for the first time that antimalarial biguanide compounds proguanil and cycloguanil also inhibit NMDA receptors in micromolar concentrations. Antiviral biguanide compound moroxydine and hypoglycemic biguanide compound metformin, in contrast, were practically ineffective. Cycloguanil acted as an NMDA receptor voltage-dependent trapping channel blocker, while proguanil and phenformin acted as allosteric inhibitors.

## 2. Results

### 2.1. Screening and Concentration Dependence

**Figure 1:**
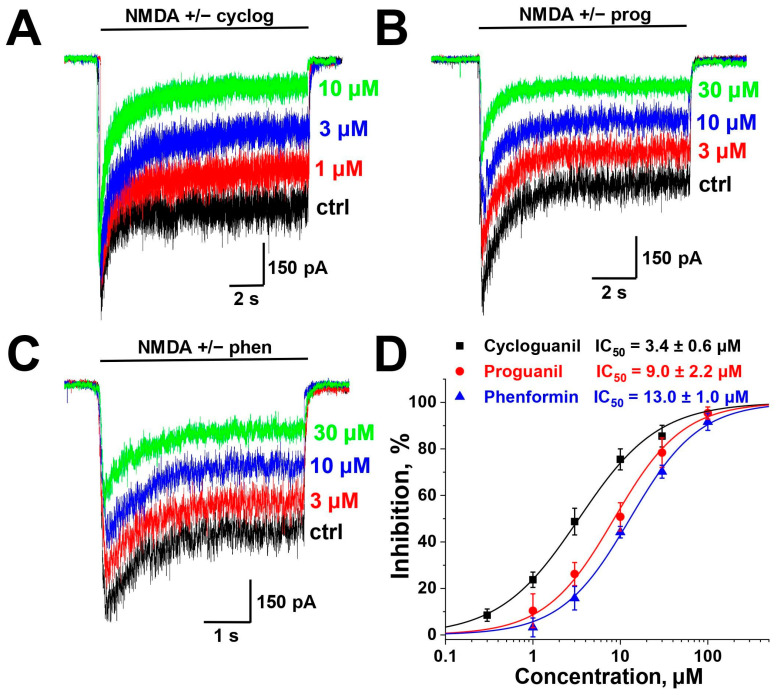
Concentration dependence of biguanide compounds action on NMDA receptors: (**A**–**C**) Representative recordings illustrating the NMDA receptors inhibition by different concentrations of cycloguanil (cyclog), proguanil (prog) and phenformin (phen), respectively. (**D**) Concentration–inhibition curves.

The application of extracellular solutions containing 100 µM NMDA and 10 µM glycine resulted in desensitizing inward currents in the pyramidal neurons of the CA1 region of the hippocampus, with a steady-state component ranging from about 300 to 1000 pA at a holding voltage of −80 mV. NMDA receptors of these neurons consist mainly of GluN1, GluN2A and/or GluN2B subunits [30]. We initially checked whether 10 µM concentrations of biguanide compounds metformin, phenformin, moroxydine, cycloguanil and proguanil would inhibit these steady-state currents. The percentages of the inhibition are presented in Table 1. Metformin and moroxydine were practically ineffective. Proguanil, cycloguanil and phenformin demonstrated significant inhibition, and we decided to study the concentration dependence of their action. Representative examples of NMDA receptor inhibition by different concentrations of biguanide compounds are demonstrated in Figure 1A–C and the concentration–inhibition curves in Figure 1D. IC_50_ values were 3.4 ± 0.6 µM for cycloguanil (*n* = 4), 9.0 ± 2.2 µM for proguanil (*n* = 5) and 13 ± 1 µM for phenformin (*n* = 5). Hill coefficients were 1.0 ± 0.1, 1.1 ± 0.2 and 1.1 ± 0.1 for cycloguanil, proguanil and phenformin, respectively.

### 2.2. Agonist Dependence

**Figure 2:**
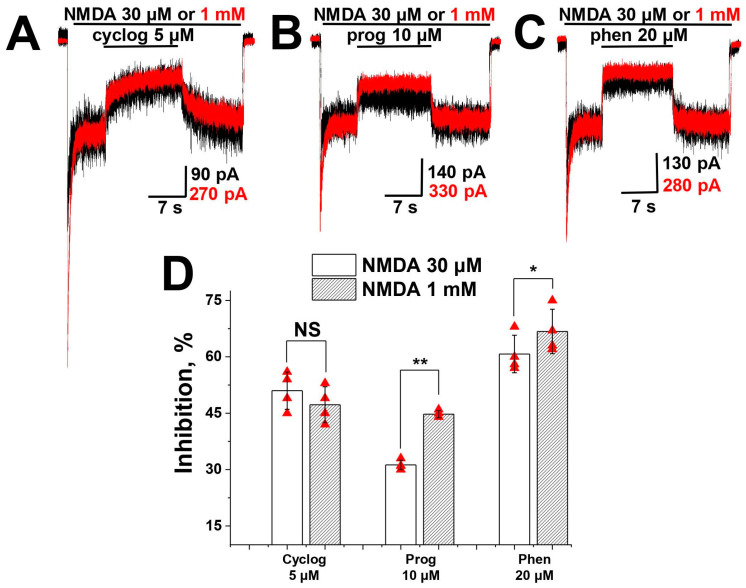
Agonist dependence of biguanide compounds action on NMDA receptors: (**A**–**C**) Representative examples of NMDA receptors inhibition by cycloguanil (cyclog) 5 µM (**A**), proguanil (prog) 10 µM (**B**) and phenformin (phen) 20 µM (**C**) at NMDA 30 µM and 1 mM. (**D**) Summary of agonist dependence data. The action of cycloguanil is agonist-independent, while proguanil and phenformin are more active in conditions of higher agonist concentration, clearly identifying that the action of all three compounds is not competitive. Paired *t*-test: NS—not significant (*p* > 0.05), *—*p* < 0.005, **—*p* < 0.001.

Ion channel inhibitors are divided into two major groups: competitive and non-competitive. To check whether NMDA receptor inhibition by proguanil, cycloguanil and phenformin is competitive or not, we compared the inhibition by these compounds at two NMDA concentrations: 30 µM (~40% of maximal response, 340 ± 120 pA, *n* = 7) and 1000 µM (~90% of maximal response, 720 ± 260 pA, *n* = 7). Representative examples for each compound and statistics are presented in Figure 2. Neither of the compounds demonstrated a decrease in activity with the increase in agonist concentration, evidencing that inhibition is not competitive. It is worth mentioning that inhibition by all amidine and guanidine compounds, studied by us earlier [28], also was not competitive.

### 2.3. Voltage Dependence

**Figure 3:**
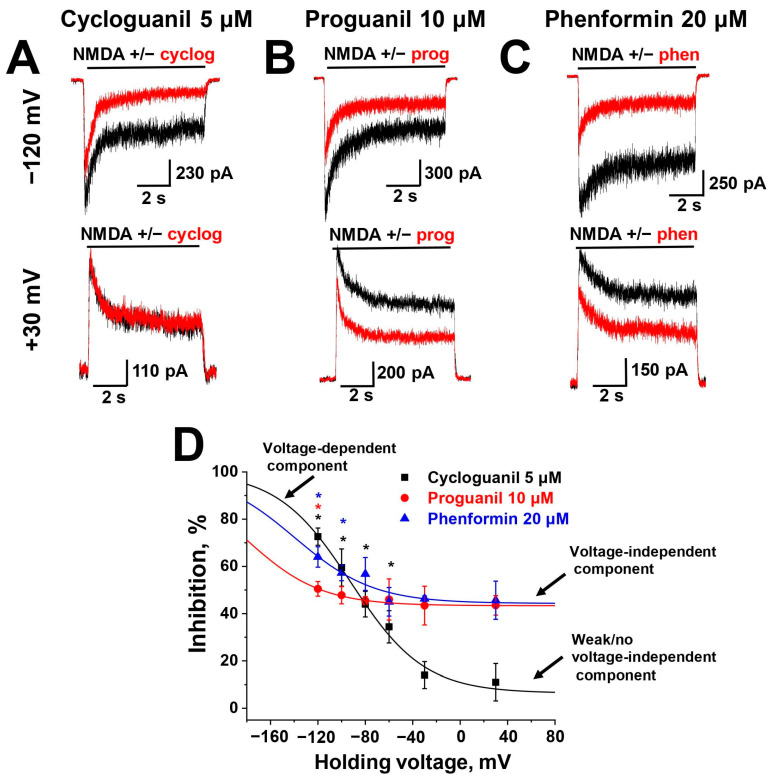
Voltage dependence of biguanide compounds action on NMDA receptors: (**A**–**C**) Representative examples of NMDA-induced currents inhibition by 5 µM cycloguanil (cyclog) (**A**), 10 µM proguanil (prog) (**B**) and 20 µM phenformin (phen) (**C**) at different holding voltages. (**D**) Summary of voltage dependence data. The data are presented as the mean ± SD. Asterisks (*) indicate the holding voltages at which the effect of cycloguanil (black asterisks), proguanil (red asterisks) or phenformin (blue asterisks) is significantly different from that at +30 mV (*p* < 0.05, *n* ≥ 4, unpaired *t*-test).

Cycloguanil, proguanil and phenformin contain biguanide groups that are positively charged at physiological pH. According to Chemaxon (https://chemaxon.com/, accessed on 5 March 2024) predictions, cycloguanil and proguanil molecules possess a +1 charge predominantly (>80%), while phenformin molecule possesses a +2 charge. This allowed for the estimation of the binding site locations within the membrane’s electrical field by examining the voltage dependence of compound action. In this series of experiments, we used 5 µM cycloguanil, 10 µM proguanil and 20 µM phenformin concentrations and changed the holding potential in the range from +30 to −120 mV. Representative examples of inhibition at +30 and −120 mV are presented in Figure 3. The inhibitory effect of cycloguanil increased with hyperpolarization, being significantly different at almost all holding voltages tested from that at +30 mV (Figure 3D, black asterisks; *p* < 0.05, *n* ≥ 4, unpaired *t*-test), suggesting that the inhibition by cycloguanil was clearly voltage-dependent. Meanwhile, the inhibition by proguanil and phenformin was significantly different from that at +30 mV only at strong hyperpolarized voltages (Figure 3D, red and blue asterisks, respectively; *p* < 0.05, *n* ≥ 4, unpaired *t*-test), suggesting that proguanil and phenformin acted mostly voltage-independently with a small voltage-dependent component. Because of the presence of the voltage-independent component, the voltage dependence curves for proguanil and phenformin were not well fitted by the Woodhull model (Equation (1), [31]). Thus, we used Equation (2), which we had elaborated on previously [28], for the fitting. Equation (2) takes both voltage-dependent and voltage-independent binding into account. The results of the fitting are presented in Table 2. Cycloguanil has a much higher affinity to the site in the channel pore (55 µM), while proguanil and phenformin have a higher affinity to the superficial site (14 and 22 µM, respectively). Such difference in the voltage-dependence of action between similar biguanide compounds is intriguing, and the underlying structural reasons are presented in the Section 3.

The parameters Kvi and Kvd represent the drug’s affinity for the superficial and deep binding sites, respectively, while the δ value indicates the fraction of the membrane electric field that the charged blocking molecule traverses when moving from the external environment to the binding site within the channel. Asterisks indicate the fixed value.

### 2.4. Competition with Magnesium for Binding Site

NMDA receptor voltage-dependent block by magnesium is critical for CNS function [32,33]. So, it is not surprising that the magnesium binding site in the channel is well characterized [34,35,36]. Indeed, mutations of pivotal asparagine residues at the N-site in the M2 pore-lining region of NMDA receptors significantly attenuate magnesium block [34]. This site in the channel pore is shared by magnesium with many other cationic channel blockers, including memantine and ketamine [37,38]. They compete with magnesium for the binding site, and their activities are reduced in the presence of magnesium [39,40]. Thus, we decided to check whether biguanide compounds compete with magnesium for binding sites.

**Figure 4:**
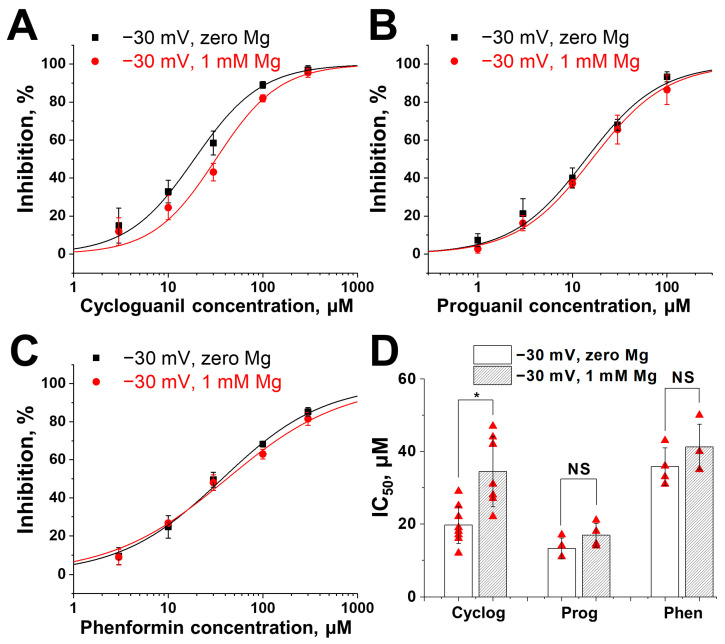
Concentration dependencies of biguanide compounds action at −30 mV holding voltage in the absence or presence of 1 mM Mg^2+^. (**A**–**C**) The activity of cycloguanil is attenuated in the presence of magnesium (**A**), while that of proguanil (**B**) and phenformin (**C**) is not affected. (**D**) Summary of data on biguanide compounds activities in the absence or presence of 1 mM Mg^2+^. Unpaired *t*-test: NS—not significant (*p* > 0.05), *—*p* < 0.005. IC_50_ value for cycloguanil (cyclog) at −30 mV holding voltage was 20 ± 5 µM (*n* = 9), while that in the presence of 1 mM Mg^2+^ was significantly higher (34 ± 10 µM, *n* = 7, *p* = 0.001, unpaired *t*-test). IC_50_ value for proguanil (prog) at −30 mV holding voltage was 13 ± 3 µM (*n* = 4), which was not significantly different from that in the presence of 1 mM Mg^2+^ (17 ± 3 µM, *n* = 4, *p* = 0.15, unpaired *t*-test). IC_50_ value for phenformin (phen) at −30 mV holding voltage was 36 ± 5 µM (*n* = 4), which was not significantly different from that in the presence of 1 mM Mg^2+^ (41 ± 6 µM, *n* = 4, *p* = 0.23, unpaired *t*-test).

At a physiological magnesium concentration of 1 mM, NMDA-induced currents are minimal at −80 mV and reach near maximum levels at −30 mV. The activities of the compounds at −30 mV holding voltage with magnesium in an external solution facilitate more accurate predictions of their effects in vivo. We compared the concentration dependencies of action of proguanil, cycloguanil and phenformin at −30 mV in the absence and presence of 1 mM magnesium. The activity of cycloguanil (Figure 4A) was significantly higher in the absence of magnesium (IC_50_ = 20 ± 5 µM (*n* = 9)) compared to 1 mM magnesium (IC_50_ = 34 ± 10 µM (*n* = 7), *p* = 0.001, unpaired *t*-test). In contrast, the IC_50_ values for proguanil and phenformin were the same in the absence and presence of magnesium at −30 mV holding voltage (Figure 4B,C). These data agree well with the strong voltage dependence of the action of cycloguanil and mostly voltage-independent inhibition of proguanil and phenformin.

**Figure 5:**
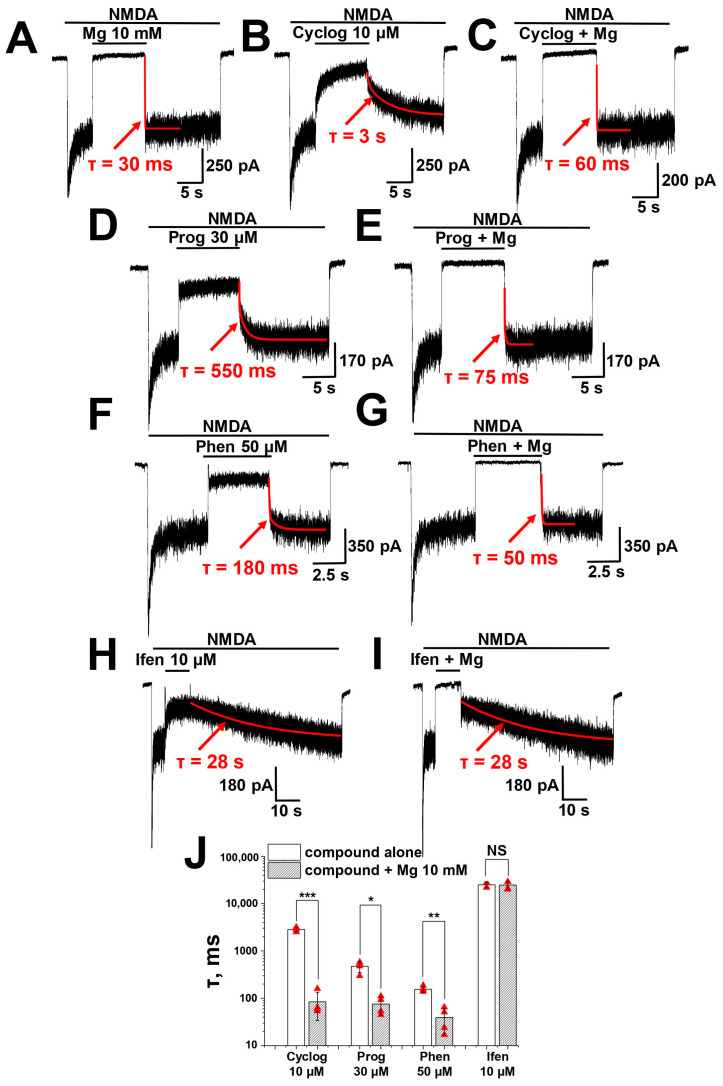
Magnesium prevents biguanide compounds from binding to NMDA receptors at −80 mV holding voltage: (**A**) Washout of Mg^2+^ 10 mM is very fast. (**B**,**C**) Washout of cycloguanil (cyclog) 10 µM is slow (**B**), while washout of cycloguanil and Mg^2+^ mixture is significantly faster (**C**). (**D**,**E**) Washout of proguanil (prog) 30 µM and Mg^2+^ mixture is significantly faster than of proguanil alone. (**F**,**G**) Washout of phenformin (phen) 50 µM and Mg^2+^ mixture is significantly faster than of phenformin alone. (**H**,**I**) In contrast, magnesium does not affect the washout kinetics of ifenprodil (ifen). (**J**) Summary of data on washout time constant (τ) for compounds alone and with Mg. Paired *t*-test: NS—not significant (*p* > 0.05), *—*p* < 0.01, **—*p* < 0.005, ***—*p* < 0.001.

At −30 mV, proguanil and phenformin do not compete with magnesium. At −80 mV, the situation may be different because of the presence of voltage-dependent components in their action. We checked it by analyzing the effect of magnesium on the washout kinetics of biguanide compounds at −80 mV (Figure 5). In this series of experiments, we used excessive concentrations of magnesium (10 mM) and moderate concentrations of biguanide compounds, producing about 80% inhibition. Washout of magnesium alone was very fast (τ = 30 ± 16 ms, *n* = 4), while those of biguanide compounds were significantly slower. Excessive concentration of magnesium practically eliminated the slow component of washout of cycloguanil, proguanil and phenformin, suggesting that the voltage-dependent component of action of all three compounds is the result of binding to the magnesium site. For instance, the washout time constant of proguanil 30 µM was 470 ± 120 ms (*n* = 4), while that of proguanil and Mg^2+^ mixture was significantly faster (τ = 76 ± 32 ms, *n* = 4, *p* = 0.005, paired *t*-test). Qualitatively similar results were obtained for cycloguanil and phenformin.

For comparison, we tested well-known allosteric GluN2B-specific NMDA receptor antagonist ifenprodil in the same protocol. As expected, the washout kinetics of ifenprodil and Mg^2+^ mixture was as slow as that of ifenprodil alone (*n* = 4, *p* = 0.76, paired *t*-test), confirming different binding sites for these two compounds and further validating our experimental protocol. A summary of data on washout kinetics is presented in Figure 5J.

### 2.5. Interaction with the Gating Mechanism of the Channel

Channel blockers are classified into two main classes according to their interaction with the channel gating mechanism. The first class is called ‘foot-in-the-door’ blockers because these compounds prevent the closure of the channel. The second class is called trapping blockers because they can remain in the channel after its closure. Among amidine and guanidine compounds, somewhat structurally similar to biguanides and studied by us earlier, nafamostat, sepimostat, diminazene and DAPI demonstrated a ‘foot-in-the-door’ mechanism, while pentamidine demonstrated partial trapping [24,27,28]. Keeping in mind that ‘foot-in-the-door’ and trapping blockers affect synaptic transmission differentially, we decided to determine the type of interaction with the channel gate for cycloguanil, proguanil and phenformin.

**Figure 6:**
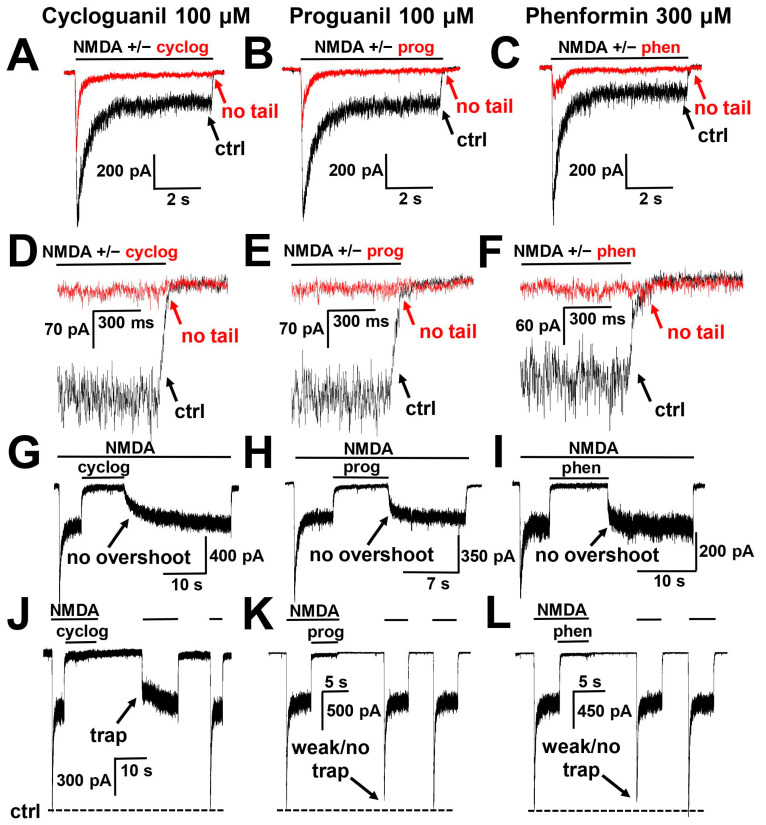
Use dependence of biguanide compounds action on NMDA receptors: (**A**–**C**) Neither cycloguanil (cyclog) 100 µM (**A**), proguanil (prog) 100 µM, nor phenformin (phen) 300 µM demonstrated tail currents. (**D**–**F**) More detailed representation of tail current absence for cycloguanil (**D**), proguanil (**E**) and phenformin (**F**). (**G**–**I**) Neither compound demonstrated overshoots in the case of washout in the presence of agonists. (**J**–**L**) Cycloguanil was significantly trapped in closed NMDA receptor channels (**J**) in contrast to proguanil (**K**) and phenformin (**L**) at −80 mV holding voltage.

The distinctive feature of ‘foot-in-the-door’ blockers is the presence of tail currents that significantly prolong the response in case of coapplication of a blocker with an agonist [15]. At −80 mV holding voltage, neither cycloguanil, proguanil, nor phenformin demonstrated them (Figure 6A–F). Another feature of ‘foot-in-the-door’ blockers is overshoot—the transient increase in the current amplitude above control levels in case of washout of a blocker in the continuous presence of agonists. In line with the absence of tail currents, cycloguanil, proguanil and phenformin did not demonstrate overshoots (Figure 6G–I). Combined, these data clearly evidence that compounds do not act as ‘foot-in-the-door’ blockers.

Finally, we analyzed the possibility of compound trapping using a double-pulse protocol [41,42], which included assessing the control NMDA response, a strong block with cycloguanil (Figure 6J), proguanil (Figure 6K) or phenformin (Figure 6L), pausing in the extracellular solution, and testing the NMDA response again. For cycloguanil, the amplitude of the testing NMDA response was significantly smaller than that of the control NMDA response, evidencing its trapping. An amount of 100 µM cycloguanil demonstrated 68 ± 11% of trapping (*n* = 6) at −80 mV holding voltage in this experiment. In contrast, proguanil and phenformin demonstrated <5% trapping in the ‘double-pulse’ protocol. In the case of these two compounds, voltage-independent allosteric inhibition dominates, and they do not demonstrate clear signs of either ‘foot-in-the-door’ or trapping channel block.

## 3. Discussion

In this paper, for the first time, we tested biguanide compounds for their ability to directly inhibit NMDA receptors. To the best of our knowledge, among biguanide compounds, the inhibitory action on NMDA receptors has only been shown previously for phenformin [29]. But, the effect was considered indirect because acute phenformin treatment did not affect NMDA-induced whole-cell current and did not alter calcium responses to NMDA, in contrast to pretreatment experiments with phenformin, in which inhibitory action was observed [29]. In our experiments on native rat hippocampal NMDA receptors, biguanide compounds moroxydine and metformin were practically ineffective, while cycloguanil, proguanil and phenformin directly inhibited NMDA-induced whole cell currents with IC_50_ values in the low micromolar range. Lee and coauthors [29] tested only 1 µM phenformin concentration while we tested the range of concentration from 1 to 100 µM, so the results do not directly contradict each other.

We also thoroughly studied molecular mechanisms of action of cycloguanil, proguanil and phenformin on NMDA receptors. Despite the structural similarity of these compounds, molecular mechanisms of NMDA receptor inhibition by them were significantly different. Cycloguanil acted as a voltage-dependent channel blocker with partial trapping, similar to clinically used memantine and ketamine. The inhibition by proguanil and phenformin was mostly voltage-independent. They did not demonstrate features of ‘foot-in-the-door’ or trapping channel block, suggesting allosteric inhibition. At −80 mV holding voltage in the absence of magnesium, the most active compound was cycloguanil (IC_50_ = 3.4 ± 0.6 µM), followed by proguanil (9.0 ± 2.2 µM) and phenformin (13 ± 1 µM). At −30 mV in the presence of physiological magnesium concentration, the order of potency was different—the most active compound became proguanil (IC_50_ = 17 ± 3 µM), followed by cycloguanil (34 ± 10 µM) and phenformin (41 ± 6 µM). In these conditions, the activity of proguanil was approximately three-fold lower than that of clinically used memantine (IC_50_= 6.4 ± 0.5 µM [40]). Compared to activities at −80 mV in the absence of magnesium, activities of all biguanide compounds at −30 mV in the presence of magnesium were reduced. The degree of decrease was much stronger for cycloguanil because of the stronger voltage dependence of its action and competition with magnesium for the binding site in the channel pore. These findings underscore the critical importance of evaluating activities against NMDA receptors in the presence of physiological magnesium concentration and at depolarized holding voltages, where the currents through NMDA receptors in the presence of magnesium are close to maximal.

**Figure 7:**
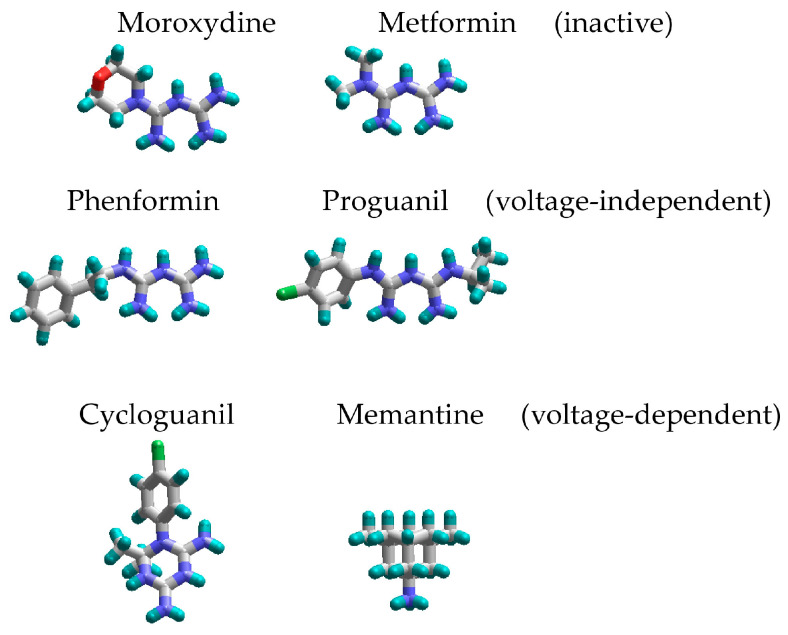
Three-dimensional structures of biguanide compounds studied. Due to the p-conjugated system, two guanidine moieties have a flat shape. Their closure into the ring in cycloguanil results in the 3D structure, which is markedly similar to the structure of memantine, a classical voltage-dependent NMDA receptor channel blocker.

In order to explain the underlying reasons for these intriguing differences between compounds with similar chemical structures, we compared their 3D structures (Figure 7). Proguanil and phenformin, which demonstrated significant voltage-independent inhibition, possess an aromatic ring. Such ring is absent in the structures of moroxydine and metformin, which were practically inactive against NMDA receptors. The structure of cycloguanil is drastically different from that of the other members of the group. It closely resembles the 3D structure of memantine (see Figure 7). Both compounds have a compact conical shape with a charged group at the vertex, as many other NMDA receptor channel blockers [42,43,44,45]. This observation readily explains the molecular mechanism of cycloguanil inhibition—voltage-dependent channel block with partial trapping—the same as that of memantine.

**Figure 8:**
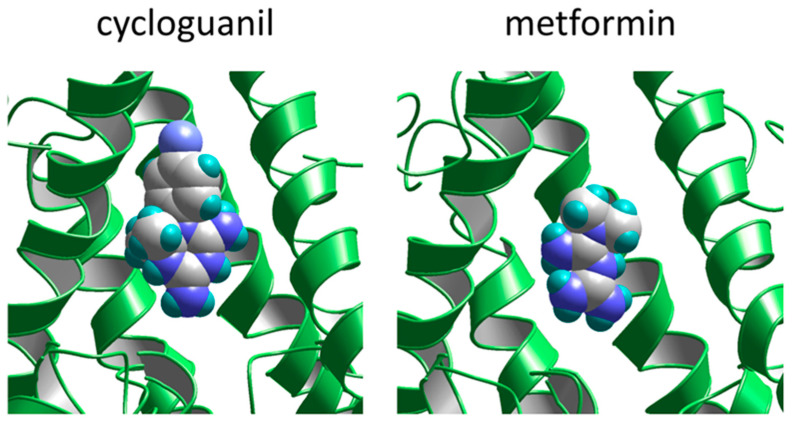
Docking of cycloguanil (**left**) and metformin (**right**) to the pore domain of the NMDA receptor. Both compounds occupy the outer vestibule just above the selectivity filter.

Finally, we decided to compare the binding of cycloguanil and metformin to the site in the channel pore using molecular docking (Figure 8). Among the compounds studied, cycloguanil has the highest affinity for this site (see Table 2), while metformin is practically inactive. The modeling was performed using ZMM software (https://www.zmmsoft.ca/, accessed on 29 August 2024) as described previously [46] with the 7sad structure of GLUN1A-GLUN2B NMDA receptor [37]. Cycloguanil and metformin were manually placed in the outer vestibule just above the selectivity filter—the known binding site of positively charged NMDA receptor channel blockers [37]. The Monte Carlo minimization procedure was used to optimize the structures and obtain the energetics of drug–channel interactions. In the optimal binding mode, both molecules are in approximately vertical orientation, with the charged group facing the selectivity filter. Despite the visual similarity, the energetics of drug–channel interactions were markedly different. The electrostatic and desolvation components of the energy were similar, while the van der Waals interactions were 8 kcal/mol more effective in the case of cycloguanil due to strong interactions of the aromatic ring (which is absent in metformin molecule) with the hydrophobic residues in M3 segments. Thus, modeling correctly reproduces the difference in activity between these compounds.

Our current work allows for the recognition of biguanide compounds as NMDA receptor inhibitors with diverse molecular mechanisms of action. It can help to repurpose some of them for the treatment of neurodegenerative diseases or depression. The present study is only the very first step on this path. Future in vitro and in vivo investigations on the possible neuroprotective effects of biguanide compounds, as well as in vivo studies in animal depression models, are needed to further evaluate the perspectives of their repurposing. It is also worth mentioning that NMDA receptors are considered potential therapeutic targets for type 1 and type 2 diabetes treatment [47,48,49,50,51]. The results from different models indicate that NMDA receptors of pancreatic islets are involved in the regulation of insulin release and blood glucose control [51]. NMDA receptor channel blocker dextromethorphan and its main metabolite, dextrorphan, demonstrated blood glucose-lowering and islet cell-protective effects both in preclinical and clinical studies [49,50]. Another structurally different NMDA receptor channel blocker, amantadine, also decreased glucose-stimulated insulin secretion from islets but was less effective as a blood glucose-lowering substance compared to the dextrorphan [51]. Finally, GluN2B-containing NMDA receptor allosteric antagonist WMS-1410 also demonstrated islet cell protective effects [47]. Taking into account these effects of NMDA receptor antagonists of different types, it is reasonable to assume that NMDA receptor inhibition by phenformin can play a role in its blood glucose-lowering and/or islet cell-protective effects.

## 4. Materials and Methods

### 4.1. Animals

All experiments were conducted with approval by the Animal Care and Use Committee of the Sechenov Institute of Evolutionary Physiology and Biochemistry of the Russian Academy of Sciences (protocol 1-13/2023, 26 January 2023). Outbred male Wistar rats (13–18 days old, weighing 25–35 g) were sourced from the IEPHB facility. Maximum efforts were made to minimize the number of animals used and to reduce discomfort.

### 4.2. Electrophysiology

The rats were decapitated after sevoflurane anaesthetization. The brains were rapidly removed and cooled to 2–4 °C. Transverse hippocampal slices were prepared using a vibratome (Campden Instruments, Loughborough, UK), and single neurons were freed from slices by vibrodissociation [52,53]. All experiments were performed at room temperature. 

The whole-cell patch clamp technique was used to record membrane currents in response to applications of the agonists. Series resistance (<20 MΩ) was compensated by 70–80% and monitored during experiments. Currents were recorded using an EPC-8 amplifier (HEKA Electronics, Lambrecht, Germany), filtered at 5 kHz, sampled and stored on a personal computer. Drugs were applied using an RSC-200 (BioLogic, Seyssinet-Pariset, France) perfusion system under computer control. The extracellular solution contained (in mM) NaCl 143, KCl 5, CaCl_2_ 2.5, D-glucose 18, and HEPES 10 (pH adjusted to 7.4 with HCl). The pipette solution contained (in mM) CsF 100, CsCl 40, NaCl 5, CaCl_2_ 0.5, EGTA 5, and HEPES 10 (pH adjusted to 7.2 with CsOH). Proguanil (HY-B0806), cycloguanil (HY-12784), phenformin hydrochloride (HY-16397A), metformin hydrochloride (HY-17471A), moroxydine hydrochloride (HY-B0420A) and ifenprodil tartrate (HY-12882A) were obtained from MedChemExpress (Monmouth Junction, NJ, USA). Other reagents were purchased from MedChemExpress (Monmouth Junction, NJ, USA), Sigma (St. Louis, MO, USA) or Tocris Bioscience (Bristol, UK). 

Experiments were performed on pyramidal neurons from the CA1 area of the hippocampus. Unless otherwise specified, NMDA receptors were activated with 100 µM NMDA and 10 µM glycine. The percentage of steady-state current blockage by various drug concentrations was measured at holding voltages of either −80 or −30 mV. Transient processes lasting more than 20 ms were modeled using single or double exponential functions. In the case of double exponential fitting, the weighted time constant was employed. 

### 4.3. Analysis of Voltage Dependence

The voltage dependence of the compounds’ action was analyzed using the classical Woodhull model [31] with the voltage-independent component addition. According to the Woodhull model of an impermeable blocker, the voltage dependence of steady-state blockade is defined by Equation (1):

(1) B=100/(1+Kb/C×exp⁡〖Fzδ/RTV〗)

where V is voltage; *B* is the level of the block (%); C is the concentration of the drug; z is the molecular charge; and R, F and T have their standard meanings. Kb is the affinity of a drug for the channel, and δ is the electrical depth of the binding site. The δ value reflects the fraction of membrane electric field that the charged blocking molecule crosses on its pathway between the external media and the binding site in the channel.

Our experimental data were not well fitted by the abovementioned equation, presumably because of the presence of a voltage-independent component. Thus, we used an Equation (2) [28], taking it into account, assuming that the binding to the deep and superficial site is independent:

(2) $$\begin{array}{r} B=100-100/(1+C/(Kvd\times exp〖Fz\delta /RTV〗)+C/Kvi\\ +(C\times C)/(Kvd\times exp〖Fz\delta /RTV〗\times Kvi)) \end{array}$$

In this equation, Kvd represents the affinity of the drug to the deep site, Kvi represents the affinity of the drug to the superficial site, and other parameters are consistent with those in Equation (1).

### 4.4. Statistical Analysis

All experimental data are presented as the mean ± SD, calculated from a minimum of four experiments (cells). The significance of the effects was tested using *t*-tests, and differences were considered significant at *p* < 0.05. Concentration dependencies were approximated using the Hill equation. In the case of voltage dependence analysis, the data from different cells (*n* ≥ 4) were pooled together and fitted with Equation (2) with the Origin 2019b 9.65 software (OriginLab Corp., Northampton, MA, USA); error values of approximation are presented as the precision measures.

## Figures and Tables

**Table 1:** Quantitative characteristics of NMDA receptor inhibition by biguanide compounds.

Compound	Chemical Structure	% of Inhibition (10 µM) −80 mV	IC_50_, µM −80 mV	Hill Coeff.
Metformin	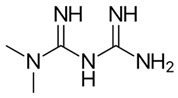	6 ± 5 (*n* = 4)	-	-
Phenformin	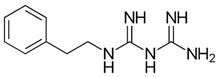	44 ± 2 (*n* = 5)	13.0 ± 1.0 (*n* = 5)	1.1 ± 0.1
Proguanil	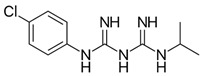	51 ± 6 (*n* = 5)	9.0 ± 2.2 (*n* = 5)	1.1 ± 0.2
Cycloguanil	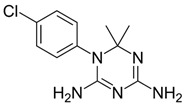	76 ± 5 (*n* = 4)	3.4 ± 0.6 (*n* = 4)	1.0 ± 0.1
Moroxydine	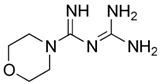	5 ± 4 (*n* = 4)	-	-

**Table 2:** Voltage dependence of NMDA receptor inhibition by biguanide compounds. *—fixed value.

Compound	Kvi, µM	Kvd, µM	δ
Cycloguanil	>500	55 ± 11	0.7 ± 0.1
Proguanil	14 ± 1	>500	0.7 *
Phenformin	22 ± 2	>500	0.7 *

## Data Availability

The data presented in this study are available on request from the corresponding author.
